# Novel Orthobunyavirus in Cattle, Europe, 2011

**DOI:** 10.3201/eid1803.111905

**Published:** 2012-03

**Authors:** Bernd Hoffmann, Matthias Scheuch, Dirk Höper, Ralf Jungblut, Mark Holsteg, Horst Schirrmeier, Michael Eschbaumer, Katja V. Goller, Kerstin Wernike, Melina Fischer, Angele Breithaupt, Thomas C. Mettenleiter, Martin Beer

**Affiliations:** Friedrich-Loeffler-Institut, Greifswald–Insel Riems, Germany (B. Hoffmann, M. Scheuch, D. Höper, H. Schirrmeier, M. Eschbaumer, K.V. Goller, K. Wernike, M. Fischer, A. Breithaupt, T.C. Mettenleiter, M. Beer);; State Veterinary Diagnostic Laboratory, Arnsberg, Germany (R. Jungblut);; Chamber of Agriculture for North Rhine-Westphalia, Bovine Health Service, Bonn, Germany (M. Holsteg)

**Keywords:** metagenomics, next generation sequencing, orthobunyavirus, Schmallenberg virus, cattle, zoonoses, viruses, Europe, Germany, the Netherlands

## Abstract

In 2011, an unidentified disease in cattle was reported in Germany and the Netherlands. Clinical signs included fever, decreased milk production, and diarrhea. Metagenomic analysis identified a novel orthobunyavirus, which subsequently was isolated from blood of affected animals. Surveillance was initiated to test malformed newborn animals in the affected region.

In summer and autumn 2011, farmers and veterinarians in North Rhine-Westphalia, Germany, and in the Netherlands reported to the animal health services, local diagnostic laboratories, and national research institutes an unidentified disease in dairy cattle with a short period of clear clinical signs, including fever, decreased milk production, and diarrhea. All classical endemic and emerging viruses, such as pestiviruses, bovine herpesvirus type 1, foot-and-mouth disease virus, bluetongue virus, epizootic hemorrhagic disease virus, Rift Valley fever virus, and bovine ephemeral fever virus, could be excluded as the causative agent. To identify the cause of the disease, we analyzed blood samples from affected cattle.

## The Study

**Figure 1:**
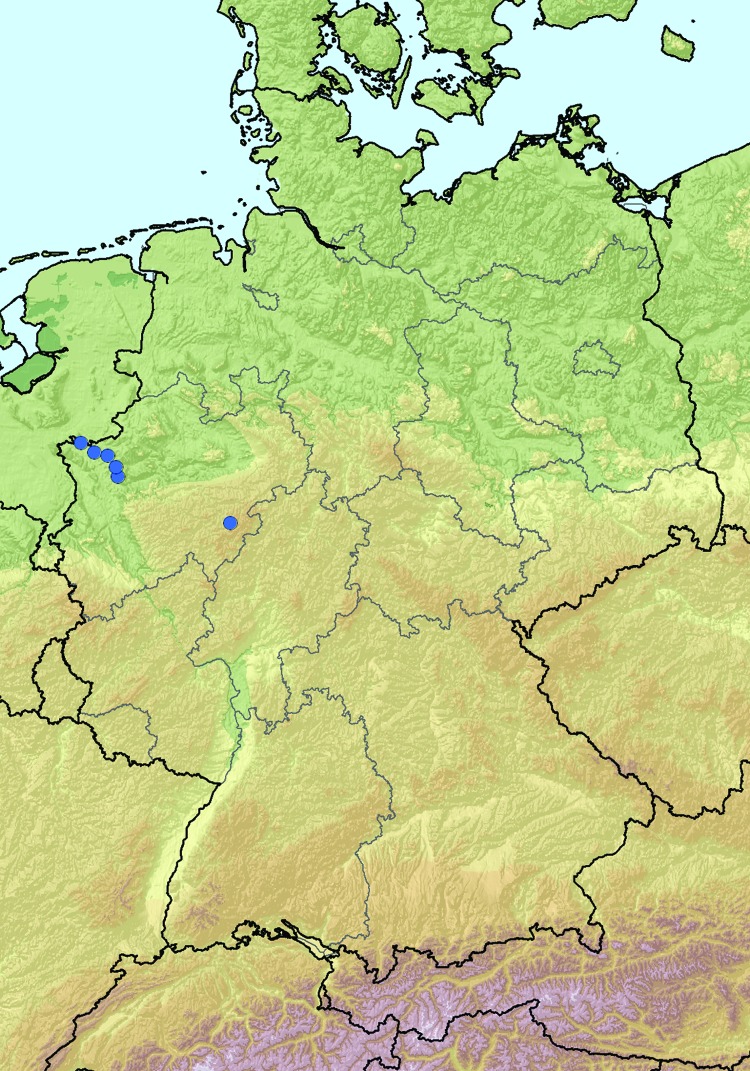
Location of farms with PCR-positive cattle (blue dots) in North Rhine-Westphalia, Germany.

On a farm near the city of Schmallenberg (North Rhine-Westphalia, Germany; Figure 1), 3 blood samples obtained in October 2011 from dairy cows that had clinical signs at sampling (Table, BH 80/11) were pooled and analyzed by using metagenomics. We also investigated a blood sample from a healthy animal from a different farm (Table, BH 81/11). For metagenomic analysis, 4 sequencing libraries (Table) were prepared and sequenced by using the 454 Genome Sequencer FLX (Roche, Mannheim, Germany). Two libraries each were generated from DNA and RNA isolated from plasma samples (Table). By using a combination of BLAST (*1*) and sequence mapping with the 454 reference mapper application (version 2.6; Roche), reads were classified into different superkingdoms (Table). In addition to the anticipated high number of host sequences, we detected in some samples a considerable portion of reads representing diverse bacterial species. These bacteria most likely grew in the samples during the prolonged storage before extraction of the nucleic acids used to prepare the sequencing libraries. Seven orthobunyavirus sequences were detected in the library prepared from pooled RNA from 3 animals of 1 farm (BH 80/11, Table). Repeated sequencing of this library resulted in 22 additional reads of orthobunyavirus-specific sequences. We assembled the reads of all 3 genome segments into contigs by using the Newbler Assembler (version 2.6; Roche). A few sequence gaps were filled by Sanger sequencing and by next-generation sequencing of the cell culture isolate. The resulting full-length sequences for the small (S; 830 nt), medium (M; 4,415 nt), and large (L; 6, 865 nt) segments are available from the International Nucleotide Sequence Database Collaboration (www.insdc.org) databases (International Nucleotide Sequence Database Collaboration accession numbers HE649912–HE649914).

**Table:** Output of raw sequence data for the sequencing libraries in the analysis of a novel orthobunyavirus in cattle, Europe, 2011

Sample	Total no. reads	No. reads classified into superkingdom					No. unclassified reads
		Eukaryota	Archaea	Bacteria	Viruses	Root	
BH 80/11 RNA (3 pooled samples)	27,413	12,296	4	13,363	55 (Myoviridae, Siphoviridae, Podoviridae, Bunyaviridae, Retroviridae, Papillomaviridae)	377	1,318
BH 81/11 RNA	16,125	10,220	2	4,821	57 (Myoviridae, Siphoviridae, Podoviridae, Retroviridae)	19	1,006
BH 80/11 DNA (3 pooled samples)	77,929	59,308	3	95	3 (Herpesviridae, Mimiviridae, unclassified virus)	9,181	9,339
BH 81/11 DNA	89,728	79,742	9	44	1 (Retroviridae)	3	9,929

Sequence comparisons were done with BLAST (*1*). The most similar sequences were from a Shamonda virus detected in cattle in Japan (S segment; INSDC accession no. AB183278; 97% identity) (*2*), an Aino virus discovered in cattle in Japan (M segment; accession no. AB542971; 71% identity) (*3*), and an Akabane virus found in cattle in Japan (L segment; accession no. AB190458; 69% identity) (*4*). This inconsistency might have resulted from the lack of published Shamonda virus M and L segment sequences and does not necessarily indicate that the novel orthobunyavirus is a reassortant. Nevertheless, only future studies that include M and L segment sequences of other members of the Simbu serogroup will enable a final classification.

**Figure 2:**
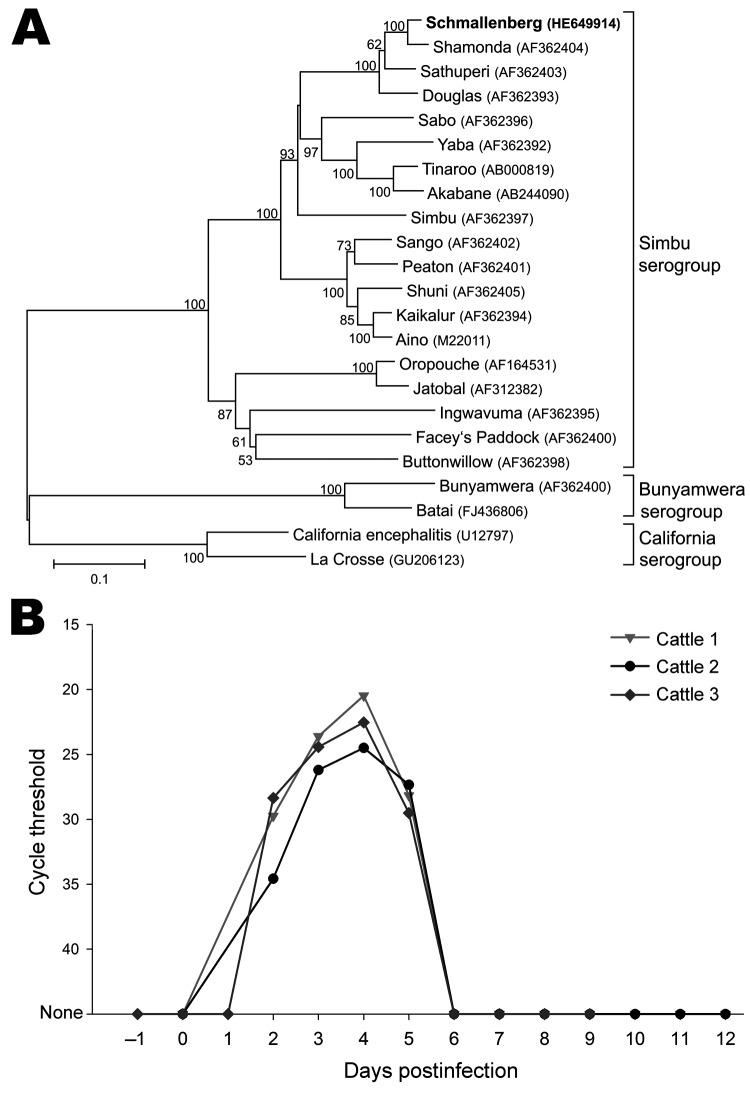
A) Phylogenetic relationship between Schmallenberg virus and orthobunyaviruses of the Simbu, Bunyamwera, and California serogroups. International Nucleotide Sequence Database Collaboration accession numbers of the sequences in the analysis are indicated in the tree. The neighbor-joining tree is based on the nucleocapsid gene of the small segment (702 nt). Numbers at nodes represent the percentage of 1,000 bootstrap replicates (values <50 are not shown). Scale bar indicates the estimated number of nt substitutions per site. B) Detection of Schmallenberg virus genome in the blood of experimentally infected calves. The highest genome copy number was detected on postinoculation day 4.

Because of the paucity of information, only S segment sequences were used for phylogenetic analysis. The S segment sequence encoding the nucleocapsid protein region (702 nt) was aligned with sequences of the Simbu, Bunyamwera, and California serogroups by using ClustalW (www.clustal.org) for codons. Phylogenetic relationship was assessed by using the neighbor-joining method based on a Tamura 3-parameter model and bootstrap analysis (1,000 replicates) as implemented in MEGA5 (*5*). The phylogenetic tree (Figure 2, panel A) shows that the S segment sequence is distinct but clusters closely with Shamonda viruses within the Simbu serogroup, which suggests that the novel virus is a Shamonda-like virus within the genus *Orthobunyavirus*.

Members of this genus within the family *Bunyaviridae* are widely distributed in Asia, Africa, and Oceania; transmission occurs predominantly through biting midges, mainly *Culicoides* spp. and mosquitoes. Especially the Simbu serogroup, which includes Akabane, Aino, and Shamonda viruses, can play a role as pathogens of ruminants. However, to our knowledge, viruses of this serogroup have not previously been detected in Europe (*6*). Because of the origin of the first positive samples, the virus was provisionally named Schmallenberg virus.

A newly developed real-time quantitative reverse transcription PCR (RT-qPCR) (primers and probes are available on request) was used to test additional samples from affected cattle farms. Twelve samples, mainly from adult cattle from 6 different farms, were positive for the novel virus, with cycle threshold (C_t_) values of 24–35. All farms with cattle that tested positive were sampled in September, October, or November and are located within the federal state of North Rhine-Westphalia. Most farms are in close proximity to the border with the Netherlands (Figure 1). The latest case from December is from a stillborn twin calf. Abdominal fluid was PCR positive for the novel virus, with a C_t_ value of ≈27.

The virus was isolated from the blood of a diseased cow from the farm in Schmallenberg. *Culicoides variipennis* larvae cells (KC cells) (*7*) (Collection of Cell Lines in Veterinary Medicine, Friedrich-Loeffler-Institut, Greifswald–Insel Riems, Germany) were incubated for 10 days with ultrasonically disrupted blood diluted in Schneider’s media. The cells were then lysed by freezing and thawing. A monolayer of baby hamster kidney-21 cells was inoculated with the lysate. The inoculum was removed after 1 h and replaced by Eagle minimal essential medium. A strong cytopathic effect was visible after 5 days, and the culture supernatant tested positive for the novel virus, with a C_t_ value of ≈14 in the specific RT-qPCR.

In a first animal trial (permit no. LALLF-7221.3–2.5–011/11) 3 calves, ≈9 months of age, were inoculated directly with blood that was PCR positive for the novel virus from 4 different cattle (1 animal was inoculated intravenously with 4 × 1 mL, 1 animal subcutaneously with 4 × 1 mL) or with the initial KC cell isolate described above (1 mL subcutaneously and 4 mL intravenously). All inoculated animals became infected and had positive PCR results 2–5 days postinoculation (dpi), with the lowest C_t_ values, ≈21, occurring at 4 dpi (Figure 2, panel B). Fever (temperature 40.5°C) developed in 1 animal 4 dpi, and 1 animal (inoculated with the KC cell isolate) had mucous diarrhea for several days. A first serum neutralization assay resulted in titers of ≈15 for serum collected 21 dpi.

## Conclusions

The detection of a novel orthobunyavirus in cattle in Germany (Schmallenberg virus) demonstrates the power of a metagenomic approach to discovering emerging pathogens. Specific and sensitive RT-qPCRs could be developed quickly and used in analyzing infected herds.

The role of the virus in the disease needs to be further investigated. However, the clinical signs in 2 of the inoculated animals, together with virus detection in samples of diseased animals in Germany and the Netherlands (*8*) and in the brain of malformed lambs in the Netherlands (*8*), strongly indicate that Schmallenberg virus caused the clinical illness. In further investigations, we will use serology to analyze distribution in the field and will sequence the complete genomes of other members of the Simbu serogroup to better understand the phylogenetic background of Schmallenberg virus.

Concern exists about the congenital defects the virus might induce in newborn calves, goats, and lambs during the next months. Therefore, surveillance has been initiated to test all malformed animals in the affected region. Some members of the Simbu serogroup, e.g., Oropouche virus, are zoonotic. However, because of the close relationship to Shamonda virus and the absence of reports of clinical signs in humans, the risk to humans currently is assessed as very low to negligible. Nevertheless, clinical and serologic surveillance in humans should be conducted in regions with infected animals to update the risk assessments.
